# Molecular Characterization of Factor VIII Gene Variants in Hemophilia A: A Genotype-Haplotype Study from Eastern and Northern-Central India

**DOI:** 10.7150/ntno.119985

**Published:** 2026-03-09

**Authors:** Chanda Hemaliya, Arun Kumar Singh, Anju Bharti, Akhtar Ali, Lalit Prashant Meena

**Affiliations:** 1 Department of General Medicine, Institute of Medical Sciences, Banaras Hindu University, Varanasi-221005, India.; 2 Department of Pathology, Institute of Medical Sciences, Banaras Hindu University, Varanasi-221005, India.; 3 Centre for Genetic Disorders, Institute of Science, Banaras Hindu University, Varanasi-221005, India.

**Keywords:** hemophilia A, Factor VIII, F8 gene, gene polymorphism, ARMS-PCR

## Abstract

**Rationale:**

Hemophilia A is an X-linked recessive bleeding disorder caused by a deficiency or dysfunction of factor VIII (FVIII), affecting approximately 1 in 5,000 males globally, including those in northern India. Accurate carrier detection is vital for genetic counseling and early diagnosis. This study aimed to assess the allele frequencies of three key intronic polymorphisms—IVS7-SNP (G/A), rs4898352 (T/A) in intron 18 (Bcl-I), and rs4074307 (C/T) in intron 19 (Hind-III)—in Indian children with Hemophilia A and evaluate their utility in carrier detection and linkage analysis.

**Methods:**

A total of 205 unrelated male children with Hemophilia A, representing 195 families from Eastern and Northern-Central India were included. Diagnosis was confirmed via FVIII assay. Genomic DNA was extracted from peripheral leukocytes. Genotyping of the three selected intronic polymorphisms in the F8 gene was carried out using ARMS-PCR.

**Results:**

The positive allele frequencies observed for IVS7-SNP, Bcl-I, and Hind-III were 0.10, 0.53, and 0.36, respectively. The negative allele of IVS7-SNP was found to be more prevalent among affected siblings. Family histories frequently revealed multiple affected individuals and hemophilia-related deaths, emphasizing the hereditary burden of the disorder in these regions.

**Conclusion:**

The distribution of the studied polymorphisms is consistent with global heterozygosity patterns and underscores their potential role in carrier screening and linkage analysis. These findings provide a feasible genetic tool for early diagnosis and counseling in Hemophilia A, particularly in low-resource settings where direct mutation analysis is limited.

## Introduction

Hemophilia A is an X-linked recessive inherited bleeding disorder caused by a deficiency or dysfunction of coagulation factor VIII (FVIII), resulting from pathogenic variants in the F8 gene. Itis the most common inherited bleeding disorder, responsible for about 80% of haemophilia cases worldwide, with an estimated incidence of approximately 1 in 5,000 male** births** 1,2. The disease predominantly affects males, while females are typically asymptomatic carriers due to the presence of a normal allele on the second X chromosome. However, symptomatic hemophilia can occur in females through homozygous or compound heterozygous mutations, or in cases of extreme lyonization 3-5. The severity of hemophilia A correlates with plasma FVIII activity levels: severe (<1% or <0.01 IU/mL), moderate (1-5% or 0.01-0.05 IU/mL), and mild (5-40% or 0.05-0.40 IU/mL) 6. Individuals with severe hemophilia A often present in early childhood with spontaneous bleeding episodes, particularly hemarthroses, which can lead to debilitating arthropathy if inadequately managed. Standard care involves prophylactic or on-demand replacement therapy using recombinant or plasma-derived FVIII products, including extended half-life formulations 7,8. The F8 gene, located on Xq28, spans 186 kb and comprises 26 exons encoding a 2,332 amino acid glycoprotein with A1-A2-B-A3-C1-C2 domains. FVIII is synthesized primarily in the endothelial cells and circulates bound to von Willebrand factor (vWF). Upon activation by thrombin, the B domain is removed, converting FVIII to its active form (FVIIIa), which acts as a cofactor to activated FIX (FIXa) in the intrinsic tenase complex, enhancing FX activation and thrombin generation 9-13. More than 3,000 mutations have been identified in the F8 gene, ranging from intron 22 and intron 1 inversions, which account for nearly half of all severe cases, to point mutations, deletions, insertions, and splice-site alterations 14,15. Genotype-phenotype correlations are important for predicting disease severity, inhibitor risk, and for providing accurate genetic counseling and prenatal diagnosis. Haplotype analysis of the F8 gene also aids in understanding mutation origins and patterns of inheritance. Although haemophilia care has advanced significantly in high-income countries, challenges persist in many low- and middle-income regions, including north and east India. Limited access to molecular diagnostics, inadequate supply of factor concentrates, and lack of population-specific mutation data contribute to suboptimal care 6.

This study aims to characterize the genotypes and haplotypes of children with haemophilia A from Northeast India, with the goal of improving diagnostic accuracy, enabling carrier detection, and informing regional strategies for genetic counseling and therapeutic planning.

## Materials and Methods

### Study Design and Participants

This study investigated marker genotypes and haplotype patterns in male patients with haemophilia A whose families were enrolled in our Day care centre of hemophilia. Clinical severity of haemophilia A was categorized based on plasma FVIII activity (FVIII:C) levels: severe (<1%), moderate (1-5%), and mild (5-40%). Among the studied families, 11 (73%) had severe haemophilia, 3 (20%) had moderate haemophilia, and 1 (7%) had mild disease. All study participants provided written informed consent after receiving genetic counseling.

### Clinical Evaluation

Clinical data were collected for 205 patients from the northern sates like up, bihar and eastern states like jharkhand India through physical examination, laboratory investigations, and review of medical histories. In some instances, complete clinical data could not be obtained due to limited documentation. Recorded clinical variables included age at the time of evaluation, age at first bleeding episode, history of joint and muscle bleeds, gum bleeding, ecchymoses, hematuria, gastrointestinal hemorrhage, intracranial bleeding, and spontaneous bleeding episodes. Based on bleeding frequency, patients were categorized into three groups: (i) ≥1 bleeding episode per month, (ii) ≥1 episode per year, and (iii) one episode every few years. Minor ecchymoses were excluded from the frequency count. Pedigree analysis was performed for all families to document the number of affected siblings, total affected members, and haemophilia-related deaths.

### Laboratory Investigations

Hematological evaluations included complete blood count (CBC), prothrombin time (PT), APTT, and specific clotting factor assays. Tests were performed using Al(OH)₃-adsorbed plasma, aged serum, and factor-deficient plasma within 4 hours of blood sample collection. FVIII testing was occasionally delayed by 1-2 days, with plasma stored at 20-30°C.

FVIII:C levels were assessed using the Bethesda assay, in which patient plasma was incubated with normal pooled plasma as a source of FVIII. Factor IX assays were also performed in all new cases, or verified in existing cases if results were unavailable or outdated (>24 hours old). All clotting studies were performed using a semi-automated clot analyzer.

### DNA Extraction and Genotyping

Genomic DNA was isolated from peripheral blood leukocytes using the standard phenol-chloroform extraction method. Three intragenic FVIII gene polymorphisms—IVS7 nt 27, BclI, and HindIII—were analyzed using the Amplification Refractory Mutation System PCR (ARMS-PCR). Each 25 μL PCR reaction included 100 ng of genomic DNA, 0.5 U of *Taq* DNA polymerase, 200 μmol/L of each dNTP, 2 mmol/L MgCl₂, 10 pmol of each primer (Primm, Italy), 0.1% spermidine (Sigma, USA), and 1× PCR buffer. Reaction conditions were optimized to ensure specific amplification of target polymorphisms.

To verify amplification, a 1000-bp fragment of the phenylalanine hydroxylase gene (GI:18765884, nt 89,108-90,107) was used as an internal control, amplified with the following primers:

- Intctl-F**:** 5'-CAAACCCAATAAGTGCATGCCTAC-3'
- Intctl-R: 5'-GGTACACGGCAAAATCCACAGC-3'

### PCR Conditions and Product Analysis

PCR cycling was initiated with denaturation at 94°C for 5 minutes, followed by 10 amplification cycles consisting of denaturation at 94°C for 1 minute, annealing at 68°C for 30 seconds, and extension at 72°C for 1 minute. A final extension step was carried out at 72°C for 7 minutes. ARMS-PCR amplification was conducted using specific primers for IVS7 nt 27, HindIII, and BclI markers. Detection sensitivity was evaluated by including known positive controls in each run. Amplified products were resolved on a 2% agarose gel stained with ethidium bromide and visualized under ultraviolet (UV) light. Gel documentation was performed to assess amplification specificity and confirm genotype identification. Multiplex ligation-dependent probe amplification (MLPA) was used to confirm complete exon deletions in samples that failed to amplify by PCR.

## Results

To genotype three intragenic polymorphisms within the *F8* gene—IVS7 nt 27 SNP, BclI, and HindIII—an efficient and rapid ARMS-PCR protocol was employed. The IVS7 nt 27 polymorphism involves a G>A substitution, which was detected using allele-specific ARMS primers. Similarly, SNPs located at the recognition sites for the restriction enzymes BclI and HindIII were analyzed, generating restriction fragment length polymorphisms (RFLPs) indicative of allelic variation within the *F8* gene.

### Molecular Characterization of Genetic Markers

The HindIII polymorphism results from a C>T substitution at the restriction site (AAGCTT → AAGTTT), located at nucleotide 4123 (GI:182375). The BclI polymorphism is caused by a T>A substitution in the recognition sequence (TGATCA → TGAACA), located at position 122021 (GI:56385011).

RFLP analysis using ARMS-PCR successfully identified all three polymorphisms in the studied population. Each PCR amplification included a 1000 bp internal control fragment from the *phenylalanine hydroxylase* gene to ensure amplification fidelity and to rule out false negatives.

**Figure 1:**
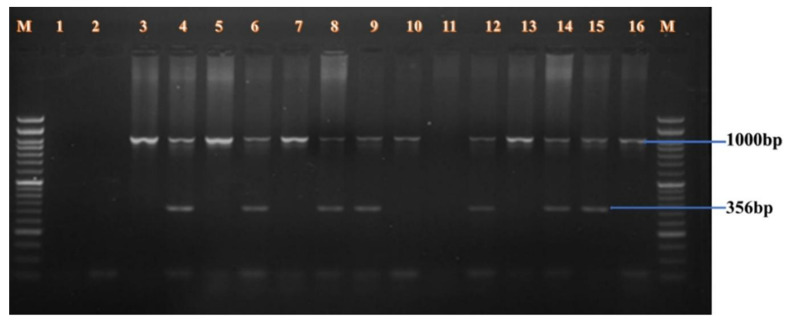
BclI Polymorphism.

**Figure 2:**
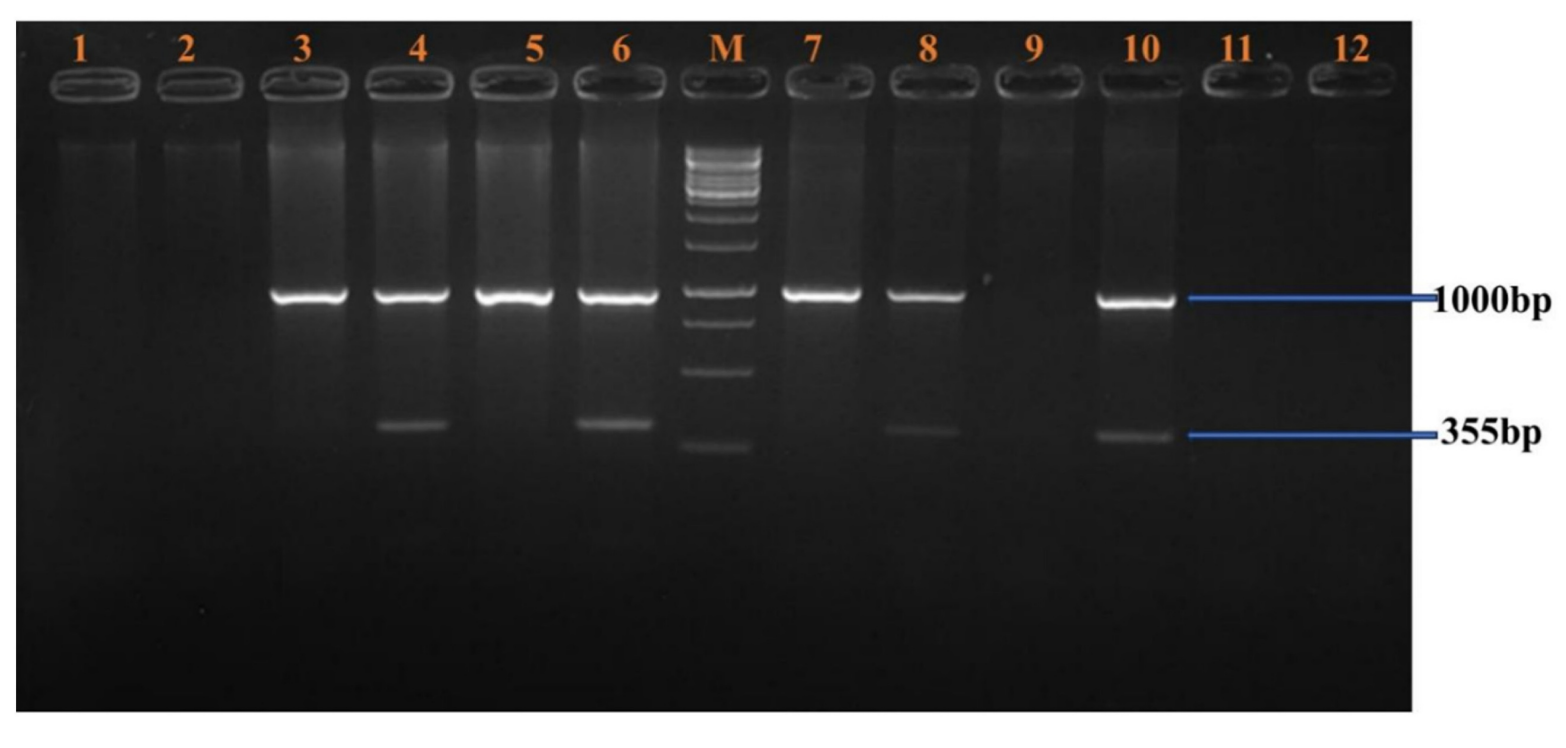
HindIII Polymorphism.

**Figure 3:**
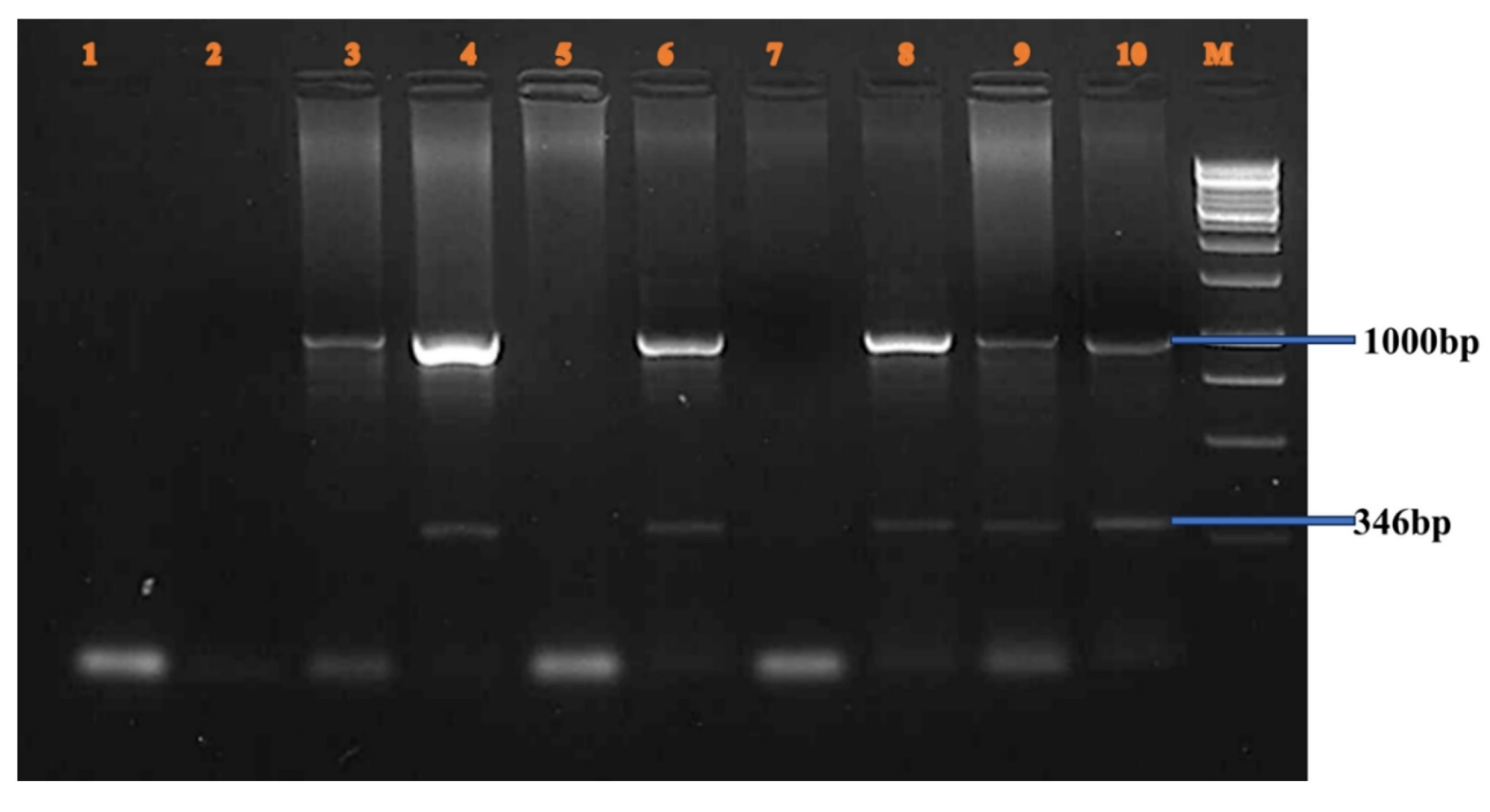
IVS7 nt 27 SNP.

- **BclI Polymorphism (Figure 1):** A 356 bp fragment from intron 18 was amplified in male samples. Lanes 3-16 showed amplification of both the polymorphic fragment and the internal control. Homozygous positive alleles (356 bp) were visualized in lanes 3-4, 5-6, 7-8, 11-12, and 13-14. Negative controls were shown in lanes 1 and 2. Lanes 9-10 and 15-16 showed undigested PCR products of 356 bp, indicating either heterozygosity or absence of restriction at the site.
- **HindIII Polymorphism (Figure 2):** A 355 bp fragment from intron 19 was amplified and resolved. Homozygous positive samples were observed in lanes 3-10. Lanes 1-2 and 11-12 served as negative controls. A molecular weight marker was loaded in lane M.
- **IVS7 nt 27 SNP (Figure 3):** A 346 bp fragment from intron 7 was amplified in female carriers. Lanes 3-10 displayed both the target fragment and the internal control. Positive homozygous G alleles were observed in lanes 3-4, 5-6, and 7-8. Lanes 9-10 also displayed 346 bp fragments, consistent with the expected ARMS-PCR amplicons.

### Genotype and Allele Frequency Analysis

Allele frequencies were calculated from the genotype distributions under the assumptions of Hardy-Weinberg equilibrium. The observed allele frequencies among hemophilia A patients from Uttar Pradesh and Bihar were:

- **BclI (T/A):** 0.46 / 0.54
- **HindIII (C/T):** 0.46 / 0.53
- IVS7 (pK1.1) (G/A): 0.10

These results suggest moderate polymorphism at the BclI and HindIII loci, and low polymorphism at IVS7 in the studied population. The observed variation provides a useful set of informative markers for indirect linkage analysis in hemophilia A families in this region.

### Application of Genotyping for Carrier Detection and Prenatal Diagnosis

All three genetic markers were successfully genotyped in 205 male haemophilia A patients from unrelated families. The ARMS-PCR technique proved to be reliable, rapid, and cost-effective for indirect carrier detection and prenatal diagnostic applications. Haplotype analysis of the three markers facilitated the establishment of marker-phase information, essential for tracking the inheritance of the defective *F8* allele in families (Table 3).

A schematic summary of the *F8* gene, showing the physical positions of the polymorphic sites and their dbSNP identifiers. This mapping provides a reference for future studies utilizing these markers in the Indian population.

## Discussion

**Figure 4:**
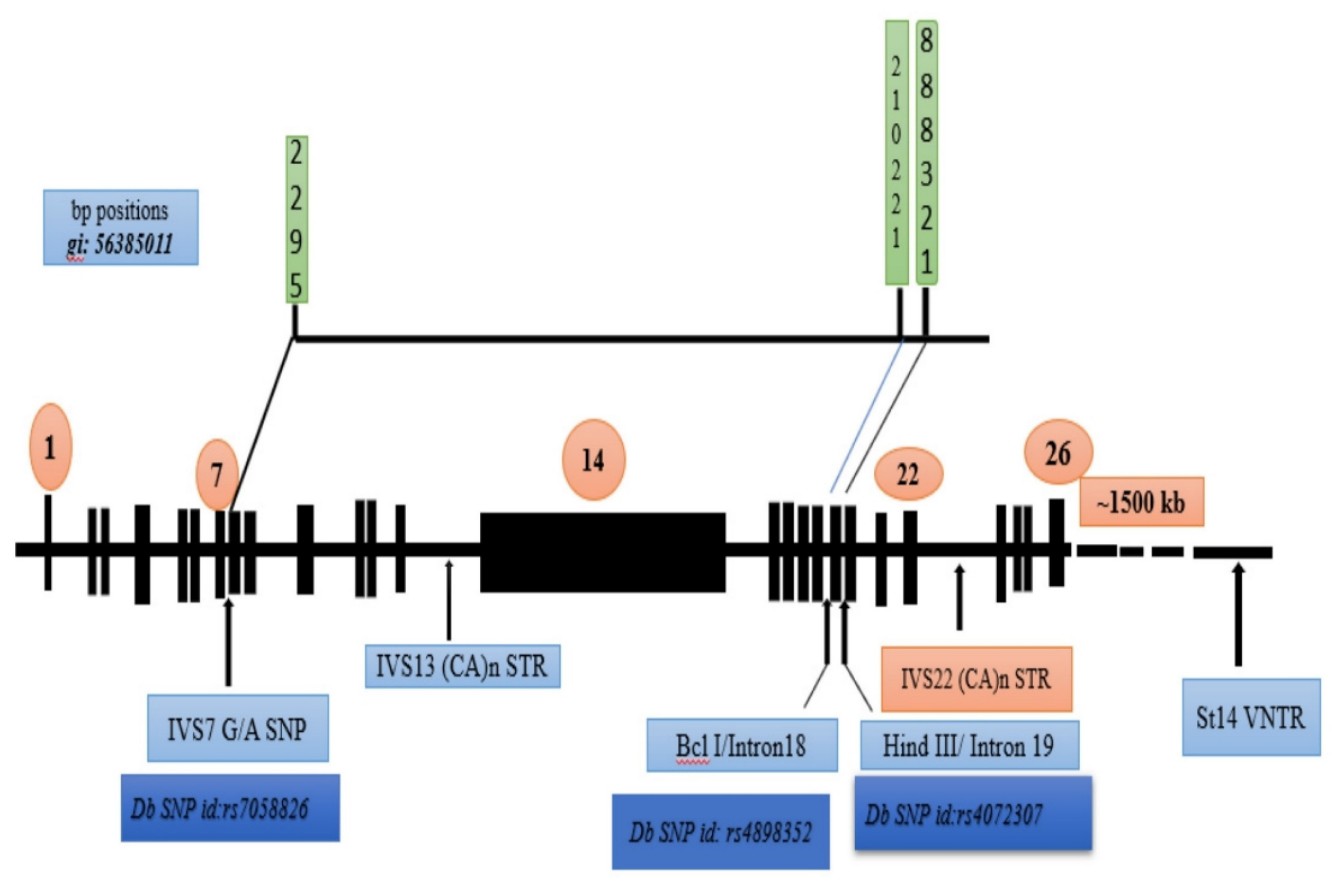
The base pair positions of the human FVIII gene, along with the corresponding dbSNP identifiers and the IVS7 nt 27 SNP, BclI, and HindIII genetic markers, were examined 31.

The clinical manifestations of hemophilia A demonstrate considerable heterogeneity, making the detection of carriers and the interpretation of genetic variations particularly challenging. Numerous studies have explored the informativeness of specific intronic markers within the *factor VIII* gene—particularly introns 7 (IVS7), 18, and 19—in the genetic diagnosis and carrier detection of hemophilia A. This study confirms and expands prior Indian data on intronic FVIII polymorphisms by analyzing the allele frequency distributions of these markers among unrelated families from Northeast India and evaluating their potential utility in genetic screening (Figure 4).

In our study cohort of 205 individuals, the positive allele frequencies for introns 7, 18, and 19 were found to be 0.10, 0.53, and 0.36, respectively. These findings closely mirror previous Indian studies, particularly with respect to intron 19, where the observed frequency (0.36) aligns with those reported by Choudhury MR *et al.* (2000) [0.35], Srinivasan A *et al.* (2002) [0.33], and Raza ST *et al.* (2009) [0.38]. The heterozygosity rate for intron 19 in our study (52%) was the highest among the three markers assessed, underscoring its polymorphic nature and potential diagnostic utility.

A comparative analysis of allele frequencies across different Indian studies (Table 4) highlights both consistencies and variations in marker informativeness. For example, while our observed intron 18 allele frequency (0.53) is lower than that reported by Srinivasan A *et al.* (2002) [0.68] and Prabhavati P *et al.* (2002) [0.67], it is closely comparable to the findings of Raza ST *et al.* (2009) [0.57] and Amit *et al.* (2018) [0.54]. These differences further confirm the polymorphic nature of these markers and suggest regional genetic variability across Indian populations.

Additionally, when compared with global data (Table 5), heterozygosity rates for intron 18 and intron 19 in our population (approximately 50%) are broadly consistent with those reported in other ethnic groups such as Caucasians, Chinese, and Malays. This reinforces the value of these intronic markers as viable tools for linkage-based diagnosis in diverse populations.

From a clinical perspective, the intron 19 negative allele showed a significant association with the presence of an affected sibling, indicating a possible role in familial transmission. Pedigrees with a higher number of hemophilia-affected members and related deaths exhibited a higher prevalence of the intron 19 negative allele, although this association did not reach statistical significance. In contrast, intron 18 alleles did not show notable trends in relation to clinical severity or familial clustering of the disease, suggesting that while intron 18 remains polymorphic, its diagnostic correlation with clinical features may be limited.

Carrier detection was notably improved through the combined use of Hind III (intron 19), and Bcl I (intron 18) markers, achieving a diagnostic yield of 87.2% in hemophiliac families. This is comparable to findings by Srinivasan A *et al.* (2002), who reported a 65% detection rate using only intron 18 and 50% using intron 19, and by Prabhavati P *et al.* (2002), who demonstrated 100% detection among 15 South Indian families using polymorphic sites in introns 18 and 22.

To confirm complete exon deletions in samples showing no amplification by PCR, multiplex ligation-dependent probe amplification (MLPA) was performed. MLPA is a robust and sensitive methodology capable of detecting gene dosage variations arising from deletions or duplications involving one or more exons 25.

### Treatment Modalities

Currently, the most promising curative approach for hemophilia A and B is gene therapy. As of June 2024, eleven clinical trials for gene therapy in hemophilia are underway, with approximately 300 participants—202 with hemophilia A and 135 with hemophilia B. A single injection of a viral vector aims to achieve sustained endogenous production of factor VIII or IX, thereby significantly reducing the bleeding risk.

Nevertheless, the success of immune tolerance induction (ITI) therapy varies between 60% and 80% depending on multiple factors, including genotype and peak inhibitor levels. High inhibitor titers in children, delayed onset of inhibitors in older patients, and previous failed ITI attempts are known risk factors for ITI failure 28,29. Moreover, ITI remains financially burdensome. Despite advances in gene therapy, challenges persist—particularly regarding genotoxicity, vector stability, immune response, and sustained gene expression. These concerns are particularly critical in pediatric patients due to evolving liver physiology and the transient nature of gene expression in developing tissues 30.

## Conclusion

Linkage analysis using polymorphic intronic markers is a well-established strategy for carrier detection and prenatal diagnosis in hemophilia A, especially when the exact mutation is unknown. In this study, three allele-specific ARMS-PCR assays were optimized to genotype markers within the *factor VIII* gene. Unlike RFLP, ARMS-PCR does not require enzymatic digestion of PCR products, making it faster, cost-effective, and suitable for clinical settings. Its application enables efficient genotyping of at-risk family members, particularly for couples seeking late-stage prenatal diagnosis where more complex techniques such as CSGE followed by direct sequencing may not be feasible.

This study reaffirms the value of intron 19 as a significant marker in familial cases of hemophilia A, whereas intron 18, despite its polymorphism, showed limited clinical correlation. Overall, the integration of multiple genetic markers enhances diagnostic accuracy and facilitates better genetic counseling and reproductive decision-making for affected families. Based on our findings, screening implementation in resource-limited settings such as in India can be strengthened by incorporating the cost-effective ARMS-PCR-based genotyping of intronic polymorphisms (IVS7, BclI, HindIII) into routine diagnostic protocols. Given the high heterozygosity rates observed for intron 18 and 19, these markers offer significant utility for indirect carrier detection and prenatal diagnosis.

## Figures and Tables

**Table 1:** Patient clinical manifestations by severity

Clinical Manifestation of Patients According to Severity (n=205)		
Severity of hemophilia	Number of patients	Percentage (%)
Mild hemophilia	21	10.0 %
Moderate	49	24.0%
Severe	135	65.9%

**Table 2:** ARMS primers

Genetic polymorphism	Primer Sequence	Amplicon Size/ Annealing temperature
IVS7SNP-G (G>A) IVS7SNP-A IVS7SNP-Com	5'-TAGAACAGCCTAATATAGCAAGACACCCTG-3' 5'- TAGAACAGCCTAATATAGCAAGACACCCTA-3' 5'-CATAGTCCCAGTCCTCCTCTTCAG-3'	265bp, 68°C for 0.30 Sec, (G allele) (A allele) (G and A allele common for both primers)
Hind III-C Hind III-T Hind III-Com	5'GGTTGCTTGTGTCAGGAAATAAATACCAGC-3' 5'-GGTTGCTTGTGTCAGGAAATAAATACCAGT-3' 5'-TCCATACATAATAGACCAGTGGCTC-3'	346 bp, 64°C for 0.30 sec, (c allele) (T allele) (C and T allele common for both primers)
Bcl I-T Bcl I-A Bcl I-Com	5'-GTCTAGGCACTGGGAACACAATCAGAGAT-3' 5'-GTCTAGGCACTGGGAACACAATCAGAGAA-3' 5'-GGATGACTACTGGTGCCCTATGG-3'	354bp, 68°C for 0.30 sec (T allele) (A allele) (T and A allele common for both the primers)

**Table 3:** Factor VIII gene allele frequency for the markers Intron 7, introns 18 and 19:

Polymorphic marker	Restriction enzyme	Number of patients	Alleles	Allele Frequency
Intron 7	IVS7-SNP	205	205	0.10
Intron 18	Bcl-I	205	205	0.53
Intron 19	Hind-III	205	205	0.36

**Table 4:** Allele frequency for the factor VIII gene's IVS7, introns 18 and 19 markers:

Study	Year	Number of cases	Intron 7 positive allele frequency	Intron 18 positive allele frequency	Intron 19 positive allele frequency
Other countries					
Howarth A (UK)	1998	105	0.88/0.12 G/A	0.80/0.20 T/A	------
Azimifar *et al.* (Iran)	2006	85	0.88/0.12 G/A	0.52/0.48 T/A	0.48/0.52 C/A
					
India					
Shetty S *et al.*	1997	37	----	0.41	----
Choudhury MR *et al.*	2000	63	---	0.43	0.35
Srinivasan A *et al.*	2002	112	---	0.68	0.33
Prabhavati P *et al.*	2002	43	---	0.67	----
Jayandharan G *et al.*	2004	50	---	---	0.29
Raza ST *et al.*	2009	100	---	0.57	0.38
Amit *et al.*	2018	59 and 62 respectively		0.54	0.34
Present Study	2024	205	0.10	0.53	0.36

**Table 5:** Heterozygosity rates for the factor VIII gene's INS7-SNP intron 18 and intron 19 markers in various ethnic groups worldwide

Ethnic group	Heterozygosity rate for IVS 7 SNP marker	Heterozygosity rate for intron 18 marker	Heterozygosity rate for intron 19 marker
Caucasian	---	0.39	0.38
Japanese	---	0.42	----
Chinese	----	0.33	0.37
Malays		0.33	0.35
American Black		0.31	0.34
